# Vegan diet and blood lipid profiles: a cross-sectional study of pre and postmenopausal women

**DOI:** 10.1186/1472-6874-14-55

**Published:** 2014-04-08

**Authors:** Yee-Wen Huang, Zhi-Hong Jian, Hui-Chin Chang, Oswald Ndi Nfor, Pei-Chieh Ko, Chia-Chi Lung, Long-Yau Lin, Chien-Chang Ho, Yi-Chen Chiang, Yung-Po Liaw

**Affiliations:** 1 Department of Psychiatry, Changhua Christian Hospital, Changhua, Taiwan; 2 Department of Public Health and Institute of Public Health, Chung Shan Medical University, No. 110 Sec. 1 Chien-Kuo N. Road, Taichung City 40201, Taiwan; 3 EBM Center & Library, Chung Shan Medical University Hospital, Taichung City 40201, Taiwan; 4 Department of Family and Community Medicine, Chung Shan Medical University Hospital, Taichung City 40201, Taiwan; 5 College of Medicine, Chung Shan Medical University, Taichung, Taiwan; 6 Department of Obstetrics and Gynecology, Chung Shan Medical University Hospital, Taichung, Taiwan; 7 Department of Health and Leisure Management, Yuanpei University, Hsinchu City 30015, Taiwan

**Keywords:** Vegan, Ovo-lacto vegetarian, HDL-C, LDL-C, Triglycerides

## Abstract

**Background:**

Vegan diet has been associated with lower risk of cardiovascular diseases and mortality, partly due to its effects on serum lipid profiles. Lipid profiles [high density lipoprotein-cholesterol (HDL-C), low density lipoprotein-cholesterol (LDL-C) and triglycerides (TG)] have not been fully elucidated either in pre and postmenopausal vegans or in ovo-lacto vegetarians. This study aimed to compare lipid profiles among vegans, ovo-lacto vegetarians and omnivores.

**Methods:**

Demographic data and lipid profiles were obtained from the 2002 Taiwanese Survey on Hypertension, Hyperglycemia and Hyperlipidemia. Multivariate linear regression analysis was used to examine factors significantly and independently associated with different categories of veganism and to estimate the β value of lipid profiles in the dietary types.

**Results:**

A total of 2397 premenopausal and 1154 postmenopausal participants who did not receive lipid lowering drugs were enrolled. Premenopausal vegans had significantly lower HDL-C and higher TG, LDL-C/HDL-C, total cholesterol (TC)/HDL-C and TG/HDL-C compared with omnivores. For postmenopausal women, vegans had lower TC while ovo-lacto vegetarians were observed with low HDL-C when compared with omnivores. Multivariate linear regression analyses showed that vegan and ovo-lacto vegetarian diets decreased HDL-C levels in premenopausal women (β = -7.63, p = 0.001 and β = -4.87, p = 0.001, respectively). There were significant associations between lower LDL-C and ovo-lacto vegetarian diets (β = -7.14, p = 0.008) and also between TG and vegan diet (β = 23.37, p = 0.008), compared with omnivorous diet. Post-menopausal women reported to have consumed either a vegan or an ovo-lacto vegetarian diet were at the risk of having low HDL-C unlike those that consumed omnivorous diets (β = -4.88, p = 0.015 and β = -4.48, p = 0.047). There were no significant changes in LDL-C in both pre and postmenopausal vegans.

**Conclusions:**

Vegan diet was associated with reduced HDL-C level. Because of its effects on lowering HDL-C and LDL-C, ovo-lacto vegetarian diet may be more appropriate for premenopausal women.

## Background

The incidence of cardiovascular disease is higher in postmenopausal women than in age-matched men and premenopausal women [1-3]. There are potentially adverse changes in reductions of high density lipoprotein-cholesterol (HDL-C) levels and elevations in total cholesterol (TC), triglycerides (TG) and low density lipoprotein-cholesterol (LDL-C) levels in menopausal women [4]. The risk of metabolic syndrome also increases in postmenopausal women [5-7]. Considerable amounts of plasma lipids and lipoproteins have been reported to lower cardiovascular disease risk.

Vegetarian diets have experienced an increase in popularity [8]. A low-fat vegetarian diet improves glycemic and lipid control [9]. The consumption of a vegan diet as well as ovo-lacto vegetarian diet lowers cardiovascular disease risk and mortality [10,11]. A hospital-based survey on healthy adults proved that, vegetarians had lower total cholesterol, LDL-C and C-reactive protein levels than omnivores [12]. A meta-analysis study of randomized controlled trials concluded that a low-fat diet is efficacious in lowering the concentrations of TC, HDL-C, and LDL-C but not TG and TC/HDL-C ratio in women [13]. Healthy postmenopausal female vegetarians not on hormone therapy had lower blood pressure, TC, LDL-C, TG, and fasting glucose levels compared with the omnivores [14].

A considerable number of Taiwanese populations consume vegetarian diets due to philosophical or health concerns rather than solely religious beliefs as indicated by the increasing numbers of restaurants in cities [15]. Local Taiwanese vegetarian diets contain soybean products as major substitute for all animal products [15,16]. Vegetarian dietary patterns may be different among different subgroups and geographical regions in Taiwan. To the best of our knowledge, the association between diets and serum lipids and lipoproteins has not been reported among free-living or in pre and postmenopausal women. This study investigated the association between different categories of diets [vegan, ovo-lacto vegetarian and omnivorous diets] and lipid profiles.

## Methods

### Health data source

Subjects were recruited from the 2002 Taiwanese Survey on Hypertension, Hyperglycemia, and Hyperlipidemia (TwSHHH) database. The survey was approved, performed and provided by the National Health Research Institutes and Bureau of Health Promotion. All subjects were given written consent prior to the study. The national-wide survey was designed to evaluate the prevalence, awareness, treatment and control of hypertension, hyperglycemia, and hyperlipidemia. Study participants were randomly selected from a subsample of the National Health Interview Survey (NHIS) conducted by the National Health Research Institutes and Bureau of Health Promotion, Taiwan in 2001. The NHIS applied a multi-staged, stratified, clustered and random sampling during the study. Details of the study design and characteristics of the study population have previously been described [17,18].

Summarily, 6592 households were selected from 1648 communities with approximately 26658 non-institutionalized residents ≧of 15 years of age. Because of financial burden of biomarker screening, one-half of the communities (824 communities with 3296 households and 10292 residents) in each stratum were randomly selected from the TwSHHH. Subjects were excluded if they had received lipid-lowering drugs. Finally, 3551 women completed the interviews, physical exams and lipid profile measurements.

### Data collection and measurements

Information on socio-demographic characteristics, such as sex, age, anthropometric, physical activity, lifestyle, menopausal status, dietary habits, physician-diagnosed diseases, and medical history were obtained through household interviews. Physical examinations were made by well-trained nurses using standardized protocols.

Hip circumference (HC) was measured at the level of the greater trochanter with an anthropometric tape. Waist circumference (WC) was measured midway between the iliac crest and the last rib. Waist to hip ratio (WHR) was computed as the ratio of WC (in centimeter) to hip (in centimeters). Because body weights were not measured during the TwSHHH survey, obesity was defined as WHR ≥ 0.85 [19]. Exercise was defined as ‘yes’ for participants who exercised at least 3 days a week (30 minutes each time) for more than 3 months, and ‘no’ for those who did not exercise regularly. Fasting venous blood samples were collected to measure plasma glucose, TG, TC, HDL-C, LDL-C, creatinine, and uric acid.

Diabetic individuals were defined as those with fasting plasma glucose ≥126 mg/dl [20], and/or were on anti-diabetic medications. Prediabetes was defined by fasting plasma glucose between 100 - 125 mg/dl [20]. In females, metabolic syndrome was defined by; WC ≥80 cm and any two of the following: (1) triglyceride > 150 mg/dl, (2) HDL-cholesterol < 50 mg/dl, (3) blood pressure ≥ 130/85 mmHg or on medication, (4) fasting plasma glucose ≧ 100 mg/dl [21].

### Dietary pattern

Adherents to vegan diet excluded egg, milk, meat, poultry, seafood and by-products of animal slaughter for more than 1 year. Ovo-lacto vegetarian diet was defined as diets in which adherents completely excluded meat and fish from their diet, but included dairy and egg products for more than 1 year; otherwise, they were considered as omnivores. An organic food diet was defined as that for which subjects consumed at least 50% of total food intake as organic food, and had been on the diet for at least one year prior to the study.

### Statistical analysis

Data analysis was conducted using the SAS software package, version 9.2 (SAS Institute, Cary, NC, USA). Continuous data were expressed as means and standard deviation. Analysis of variance was used to compare the mean difference of demographic data, biochemical measurements and anthropometric indices among vegans, ovo-lacto vegetarians and omnivores. Multiple linear regressions were used to estimate the effects of the diets on lipid profiles. A *p* value <0.05 was considered statistically significant.

## Results

In total, 2397 premenopausal and 1154 postmenopausal women were enrolled. The baseline descriptive characteristics of each dietary group of premenopausal women are presented in Table 1. About 1.5% of the premenopausal were on vegan diet. Premenopausal vegans were older and had higher serum TG. Vegans and ovo-lacto vegetarians both had significantly lower HDL-C compared with the omnivores. Vegan had higher TC/HDL-C, TG/HDL-C and LDL-C/HDL-C ratios than the omnivores. There were no significant differences in WC, HC, WHR, blood pressure, fasting glucose, LDL-C and TC among the 3 dietary groups.

**Table 1:** Baseline descriptive characteristics of each dietary group of premenopausal women

**Variable**	**Vegans(n** **=** **36)**	**Ovo-lacto vegetarians(n** **=** **76)**	**Omnivores(n** **=** **2,285)**	***P-*** **value**
Age (yrs)	41.1 ± 7.2	37.6 ± 8.6	34.0 ± 10.3^ac^	<0.001^*^
WC (cm)	72.2 ± 11.0	72.7 ± 8.7	72.8 ± 9.5	0.931
HC (cm)	93.6 ± 11.8	95.8 ± 8.6	95.1 ± 8.0	0.421
WHR	0.77 ± 0.05	0.76 ± 0.05	0.76 ± 0.06	0.636
SBP (mmHg)	105.4 ± 15.2	106.7 ± 14.2	105.4 ± 13.5	0.692
DBP (mmHg)	69.3 ± 10.5	69.0 ± 10.2	69.7 ± 10.0	0.831
FPG (mg/dL)	86.6 ± 6.0	87.1 ± 19.0	89.1 ± 21.3	0.582
UA (mg/dL)	4.9 ± 1.3	4.9 ± 1.3	5.4 ± 1.4^c^	0.003^*^
Creatinine (mg/dL)	0.7 ± 0.1	0.7 ± 0.1	0.8 ± 0.1	0.216
HDL-C (mg/dL)	52.3 ± 12.4	54.9 ± 13.1	58.5 ± 13.2^ac^	0.002^*^
LDL-C (mg/dL)	111.3 ± 26.8	102.2 ± 20.9	107.7 ± 24.1	0.099
APO-A1 (mg/dL)	141.6 ± 21.4	142.4 ± 18.8	148.1 ± 23.5	0.048^*^
APO-B (mg/dL)	83.4 ± 19.4	75.9 ± 21.6	78.8 ± 21.4	0.230
TG (mg/dL)	126.3 ± 69.2	103.4 ± 42.3	97.6 ± 59.1^a^	0.012^*^
TC (mg/dL)	173.4 ± 32.5	165.8 ± 31.1	175.2 ± 33.2	0.051
TC/HDL-C	3.48 ± 1.03	3.11 ± 0.58	3.10 ± 0.78^a^	0.015^*^
LDL-C/HDL-C	2.25 ± 0.79	1.94 ± 0.51	1.93 ± 0.62^ab^	0.009^*^
TG/HDL-C	2.83 ± 2.46	2.01 ± 1.00	1.86 ± 1.79^a^	0.005^*^
Oral contraceptives use				0.557
Current	1 (2.8%)	2 (2.6%)	26 (1.1%)	
Former	3 (8.33%)	9 (11.8%)	309 (13.5%)	
Never	32 (88.9%)	65 (85.5%)	1950 (85.3%)	

APO-A1:Apolipoprotein A1; APO- B: Apolipoprotein B; DBP: Diastolic blood pressure; FPG: Fasting plasma glucose; HC: Hip Circumference; HDL-C:High density lipoprotein-cholesterol; LDL-C:Low density lipoprotein-cholesterol; SBP: Systolic blood pressure; TC: Total cholesterol; TG: Triglycerides; WC: Waist Circumference; WHR: Waist to hip ratio; UA: Uric acid.

Values are presented as mean ± S.D.

^*^Significant difference for diet type, *P* < 0.05 (ANOVA test).

^a^Significantly different from Vegans and Omnivores.

^b^Significantly different from Vegans and Ovo-lacto Vegetarians.

^c^Significantly different from Ovo-lacto Vegetarians and Omnivores.

About 5.5% of the postmenopausal women lived on vegan diet (Table 2). Vegans and ovo-lacto vegetarians and omnivores had many similarities such as age, WC, HC, WHR, blood pressure, glucose, LDL-C, Apolipoprotein-B, Apolipoprotein-A1, and TG. Vegans had lower TC while ovo-lacto vegetarians had low HDL-C when compared with omnivores.

**Table 2:** Baseline descriptive characteristics of each dietary group of postmenopausal women

**Variable**	**Vegans(n** **=** **63)**	**Ovo-lacto vegetarians(n** **=** **51)**	**Omnivores(n** **=** **1,040)**	***P-*** **value**
Age (yrs)	62.1 ± 10.0	62.8 ± 9.7	62.2 ± 10.5	0.910
WC (cm)	80.1 ± 8.5	81.2 ± 9.6	81.8 ± 9.9	0.383
HC (cm)	95.7 ± 7.2	98.8 ± 8.5	97.8 ± 8.7	0.113
WHR	0.84 ± 0.07	0.82 ± 0.05	0.84 ± 0.07	0.294
SBP (mmHg)	126.6 ± 18.8	126.3 ± 16.1	127.2 ± 20.3	0.936
DBP (mmHg)	77.0 ± 9.9	76.3 ± 10.3	77.8 ± 10.6	0.508
FPG (mg/dL)	99.6 ± 30.6	108.6 ± 40.1	103.9 ± 37.0	0.451
UA (mg/dL)	5.8 ± 1.5	6.0 ± 1.7	6.0 ± 1.7	0.676
Creatinine (mg/dL)	0.8 ± 0.2	0.8 ± 0.1	0.9 ± 0.6	0.286
HDL-C (mg/dL)	56.5 ± 11.6	55.4 ± 14.8	61.0 ± 16.1^c^	0.007^*^
LDL-C (mg/dL)	121.4 ± 28.9	128.9 ± 37.5	129.3 ± 27.4	0.107
APO-A1 (mg/dL)	155.7 ± 21.1	151.3 ± 18.8	157.4 ± 27.4	0.293
APO-B (mg/dL)	94.4 ± 26.2	97.7 ± 32.1	101.2 ± 25.1	0.096
TG (mg/dL)	140.8 ± 65.2	162.2 ± 103.6	141.1 ± 84.5	0.245
TC (mg/dL)	191.3 ± 35.4	198.9 ± 51.5	204.8 ± 38.3^a^	0.023^*^
TC/HDL-C	3.50 ± 0.89	3.74 ± 1.00	3.54 ± 1.03	0.417
LDL-C/HDL-C	2.23 ± 0.67	2.43 ± 0.73	2.26 ± 0.77	0.326
TG/HDL-C	2.78 ± 2.03	3.41 ± 3.05	2.68 ± 2.47	0.140

APO-A1:Apolipoprotein A1; APO- B: Apolipoprotein B; DBP: Diastolic blood pressure; FPG: Fasting plasma glucose; HC: Hip Circumference; HDL-C:High density lipoprotein-cholesterol; LDL-C:Low density lipoprotein-cholesterol; SBP: Systolic blood pressure; TC: Total cholesterol; TG: Triglycerides; WC: Waist Circumference; WHR: Waist to hip ratio; UA: Uric acid.

Values are presented as mean ± S.D.

^*^Significant difference for diet type, *P* < 0.05 (ANOVA test).

^a^Significantly different from Vegans and Omnivores.

Significantly different from Vegans and Ovo-lacto Vegetarians.

^c^Significantly different from Ovo-lacto Vegetarians and Omnivores.

After adjusting for potential confounders, vegan and ovo-lacto vegetarian diets had significant effects on HDL-C (β value = -7.63, p = 0.001 and β value = -4.87, p = 0.001, respectively) (Table 3). Significant associations were observed between ovo-lacto vegetarian diet and LDL-C (β = -7.14, p = 0.008) and also between vegan diet and TG (β = 23.37, p = 0.008) unlike omnivorous diet. A negative association was found between TG and exercise (β value = -10.19, p < 0.001). Current use of contraceptives was attributed to the increased LDL-C (β value = 11.28, p = 0.008) and TG (β value = 24.39, p = 0.011).

**Table 3:** Multivariate analysis of influential factors on serum lipids in premenopausal women

**Variable**	**HDL-C**		**LDL-C**		**TG**	
	***ß***	***P-*** **value**	***ß***	***P-*** **value**	***ß***	***P-*** **value**
Diet patterns						
Omnivores diet	-	-	-	-	-	-
Ovo-lacto vegetarian diet	-4.87	0.001	-7.14	0.008	6.30	0.295
Vegan diet	-7.63	0.001	0.18	0.964	23.37	0.008
Exercise habits						
No	-	-	-	-	-	-
Yes	0.77	0.277	-2.03	0.106	-10.19	<0.001
Oral contraceptives						
Never	-	-	-	-	-	-
Former	-0.32	0.693	1.61	0.260	-0.51	0.874
Current	3.61	0.130	11.28	0.008	24.39	0.011
Organic diet	1.35	0.632	-3.18	0.527	-0.89	0.937
Age (year)	0.20	<0.001	0.56	<0.001	0.70	<0.001
Hypertension	0.49	0.721	3.69	0.133	-0.76	0.890
Prediabetes^#^	-0.07	0.947	-0.65	0.745	-3.44	0.443
Diabetes	4.53	0.035	19.51	<0.001	41.03	<0.001
Metabolic syndrome	-13.25	<0.001	9.26	<0.001	98.98	<0.001
Obesity*	-0.79	0.482	3.15	0.117	6.22	0.168
Anti-gout medication	9.34	0.208	13.98	0.290	44.24	0.136

HDL-C:High density lipoprotein-cholesterol; LDL-C:Low density lipoprotein-cholesterol; TG: Triglycerides.

*Obesity is defined as waist to hip ratio ≧ 0.85.

^#^Prediabetes is defined as fasting blood sugar 100–125 mg/dl.

The associations between lipid profiles, lifestyle and other variables as assessed by multiple regression analyses are presented in Table 4. Vegan, ovo-lacto vegetarian and organic diets were significantly associated with lower HDL-C concentrations (β value = -4.88, p = 0.015; β value = -4.48, p = 0.047; β value = -11.45, p = 0.025, respectively). There were significant associations between LDL-C levels and current use of hormone replacement therapy (β values = -6.39, p = 0.017).

**Table 4:** Multivariate analysis of influential factors on serum lipids in postmenopausal women

**Variable**	**HDL**		**LDL**		**TG**	
	***ß***	***P-*** **value**	***ß***	***P-*** **value**	***ß***	***P-*** **value**
Diet patterns						
Omnivores diet	-	-	-	-	-	-
Ovo-lacto vegetarian diet	-4.48	0.047	-1.82	0.662	15.22	0.184
Vegan diet	-4.88	0.015	-6.95	0.060	4.18	0.681
Exercise habits						
No	-	-	-	-	-	-
Yes	1.76	0.083	0.08	0.967	-3.96	0.442
Hormone replacement therapy						
Never	-	-	-	-	-	-
Former	-0.42	0.768	4.48	0.090	-11.06	0.127
Current	-0.24	0.868	-6.39	0.017	4.39	0.551
Organic diet	-11.45	0.025	-3.98	0.672	-6.45	0.803
Age (year)	-0.003	0.952	-0.04	0.642	-0.10	0.677
Hypertension	-1.09	0.301	0.44	0.820	-1.04	0.846
Prediabetes^#^	0.74	0.540	4.43	0.048	-6.24	0.311
Diabetes	1.41	0.353	9.21	0.001	30.23	<0.001
Metabolic syndrome	-10.84	<0.001	6.64	0.003	72.74	<0.001
Obesity*	-1.93	0.070	-3.86	0.050	2.84	0.600
Anti-gout medication	3.76	0.156	9.01	0.066	7.42	0.582

HDL-C:High density lipoprotein-cholesterol; LDL-C:Low density lipoprotein-cholesterol; TG: Triglycerides; UA: uric acid.

*Obesity is defined as waist to hip ratio ≧ 0.85.

^#^Prediabetes is defined as fasting blood sugar 100–125 mg/dl.

## Discussion

Associations between vegetarian diets and lipid profiles in pre and postmenopausal women were found, to be independent of co-morbidities and lifestyle factors. Vegan and ovo-lacto vegetarian diets significantly lowered HDL-C concentrations in both pre and postmenopausal women as assessed by multivariate linear regression analysis. Significant association was found between LDL-C and ovo-lacto vegetarian diets in premenopausal women.

TC/HDL-C, LDL-C/HDL-C and TG/HDL-C have been reported as better predictors for cardiovascular disease risk in women [22,23]. Lower HDL-C and higher TC/HDL-C, LDL-C/HDL-C and TG/HDL-C appear unfavorable for premenopausal vegans in terms of vascular protection compared to omnivores. Reduction in LDL-C levels has been associated with favorable effects on reduction in cardiovascular disease events [24]. For premenopausal ovo-lacto vegetarians, the reduction in HDL-C was of similar magnitude as the LDL-C concentration. Ovo-lacto vegetarian diet may be more beneficial for premenopausal vegans.

In Barnard et al’ s report, a low-fat vegetarian diet led to rapid and sizable reductions in serum LDL-C (-17 mg/dl) and HDL-C (-8 mg/dl) in healthy premenopausal women, and increase in serum TG (16 mg/dl) [25]. In a hospital-based study, 35 healthy-postmenopausal ovo-lacto vegetarians without diabetes and hormone replacement therapy had significantly lower TC (mean difference: -29.6 mg/dl), LDL-C (mean difference: -24.3 mg/dl) and TG (mean difference: -30 mg/dl) compared with the age-matched omnivores [14]. A study on long-term ovo-lacto vegetarian diet in postmenopausal women without diabetes, thyroid disease and hormone replacement therapy reported that serum LDL-C (mean difference: -24 mg/dl) and TG (mean difference: -30 mg/dl) were significantly lower than those in age-matched omnivores [26]. A survey on 57 healthy postmenopausal vegans without diabetes, hyperlipidemia and hypertension had lower HDL-C (mean difference: -7.7 mg/dl), LDL-C (mean difference: -15.5 mg/dl) and TC (mean difference: -23.2 mg/dl) than those in the matched omnivores [27]. Lower TC (mean difference: -17.8 mg/dl) and borderline higher TG (mean difference: 19.5 mg/dl) were also found in a study with 102 Taiwanese Buddhist nuns [28]. Results from similar studies were considerably significant nonetheless, our study made use of a large scale population size.

In the present study, significant associations were found between vegetarian diets and lipids profiles in premenopausal than postmenopausal women. To our knowledge, no study has assessed the effects of diets on serum lipid profiles in free-living, pre and postmenopausal women. Many factors, such as lifestyle, exercises, medication and co-morbidities that may influence lipid measurements were adjusted to reduce confounding and to increase the validity of the results. There were several limitations in this study. First, TwSHHH was a cross-sectional study that may have limited the causal inference. Second, the questionnaires did not contain amount of diet and the recommended daily dietary intake. Because the results could have been affected by the quantity of diets consumed by participants, it was difficult to fully clarify the relationship between vegetarian diets and cardio-metabolic profiles. Third, a potential self-selection effect may have had an impact on our results because of certain health concerns. We reduced the confounding by adjusting hormone replacement therapy, age, organic food consumption and exercise.

## Conclusions

Vegan and ovo-lacto vegetarian diets resulted in a significant decrease in the levels of HDL-C in pre- and postmenopausal women. Lower levels of LDL-C as registered in premenopausal women could minimize possible cardiovascular disease risks attributed to lower levels of HDL-C. Ovo-lacto vegetarian diet may be more appropriate for premenopausal women. The present results cannot directly address biologic and nutritional mechanisms underlying findings; further studies should investigate such mechanisms.

## Abbreviations

HC: Hip circumference; HDL-C: High density lipoprotein-cholesterol; LDL-C: Low density lipoprotein-cholesterol; NHIS: National Health Interview Survey; TC: Total cholesterol; TG: Triglyceride; TwSHHH: The Taiwanese Survey on Prevalence of Hyperglycemia, Hyperlipidemia, and Hypertension; WC: Waist circumference; WHR: Waist to hip ratio.

## Competing interests

The authors declare that they have no competing interests.

## Authors’ contributions

YPL and YWH designed the study and revised the draft. HCC, PCK and CCH extracted and analyzed data. ZHJ, YCC and CCL interpreted data and wrote the draft. LYL and ONN provided conceptual input and contributed to the final manuscript. All authors read and provided feedback on the draft versions of the article. All the authors have read and approved the final version.

## Pre-publication history

The pre-publication history for this paper can be accessed here:

http://www.biomedcentral.com/1472-6874/14/55/prepub
